# Application of Cut-and-Sew Technique in Thoracoscopic Minimally Invasive Mitral Valve Surgery and Concomitant Maze Procedure

**DOI:** 10.21470/1678-9741-2022-0456

**Published:** 2023-11-08

**Authors:** Erlei Han, Zhifang Liu, Bing Zhou, Shuwei Wang, Zhibin Hu, Yong Cui

**Affiliations:** 1 Department of Cardiovascular Surgery, Heart Center, Zhejiang Provincial People’s Hospital, Affiliated People’s Hospital, Hangzhou Medical College, Hangzhou, Zhejiang, People’s Republic of China

**Keywords:** Mitral Valve, Maze Procedure, Electric Countershock, Freezing, Constriction, Atrial Fibrilation, Heart Valve Diseases

## Abstract

**Introduction:**

Atrial fibrillation is one of the common complications of mitral valve disease. Currently, in the absence of freezing equipment, it’s still impossible to fully conduct a minimally invasive Cox-maze IV procedure to treat atrial fibrillation.

**Methods:**

We analyzed the clinical data of 28 patients who underwent thoracoscopic minimally invasive mitral valve full maze surgery in our hospital from October 2021 to September 2022; 13 patients were male and 15 were female, three suffered from paroxysmal atrial fibrillation, and 25 suffered from permanent atrial fibrillation; average age was 61.88±8.30 years, and mean preoperative left atrial diameter was 47.12±8.34 mm. Isolation of left atrial posterior wall (box lesion) was completed in all patients by cut-and-sew technique and bipolar clamp ablation.

**Results:**

For these subjects, the median cardiopulmonary bypass time was 169 (109.75-202.75) minutes, aortic cross-clamping time was 106 (77.75-125.50) minutes, and ventilator assistance time was 6.5 (0-10) hours. Among them, eight subjects had the endotracheal tubes removed immediately after surgical operation. Three subjects were in the blanking period; two subjects still had atrial fibrillation at three months after operation, one of whom resumed sinus rhythm after electrical cardioversion therapy; and all the remaining 23 subjects had sinus rhythm.

**Conclusion:**

The minimally invasive cut-and-sew technique for electrical isolation of left pulmonary veins can improve sinus conversion rate of patients suffering from both mitral valve disease and atrial fibrillation. In selected subjects, it is safe and has good results in the short-term postoperative period.

## INTRODUCTION

**Table:** 

Abbreviations, Acronyms & Symbols	
AF	= Atrial fibrillation
IQR	= Interquartile range
PVI	= Pulmonary vein isolation

Atrial fibrillation (AF) is one of the common complications of mitral valve disease. About 1/3 of mitral valve patients suffer from AF before operation^[1-3]^. Maze surgery is regarded as the golden standard surgery to treat AF, both low-risk and high-risk patients can benefit from resuming sinus rhythm, and the guidebooks all recommend that mitral valve operation should be carried out simultaneously with AF ablation^[1,4,5]^. In China, every year, there are about 70,000 mitral valve surgical patients, < 10% of them undergo simultaneous surgical AF treatment^[1]^. One important observation is the generalization of minimally invasive surgery; currently, the absolutely majority of mitral valve surgeries are operated with minimally invasive approaches, however, minimally invasive maze procedure is still very difficult. In the lack of freezing equipment, surgeons often have to apply monopolar ablation to complete the left-sided pulmonary vein isolation (PVI) ablation, and current evidences show that monopolar ablation is inferior to the bipolar clamp or cut-and-sew approach in terms of transmurality^[1,4,6]^. Therefore, it’s essential to explore an effective maze procedure based on mitral valve surgery; before this, we have gained a rich experience in minimally invasive right infra-axillary thoracotomy^[7,8]^. After exploration and trying, we have successfully performed cut-and-sew technique in a minimally invasive right infra-axillary mitral valve surgery and concomitant maze procedure in 28 patients.

## METHODS

Since the first minimally invasive mitral valve surgery and concomitant maze procedure in October 2021 and till September 2022, we have completed 28 operations; 13 patients were male and 15 were female, three suffered from paroxysmal AF, and 25 suffered from permanent AF; average age was of 61.88±8.30 years, and mean preoperative left atrial diameter was 47.12±8.34 mm. Mitral valve disease pending surgery was confirmed in all patients by echocardiography, and AF was confirmed by electrocardiogram. All patients underwent minimally invasive right infra-auxiliary mitral valve replacement/repair and concomitant maze procedure, and the maze procedure applied was left PVI with cut-and-sew technique and bipolar clamp, while five patients also underwent tricuspid valve repair simultaneously.

All patients underwent a single-lumen tracheal intubation under general anesthesia. We cushioned each patient’s right shoulder higher for 30°, made a right infra-axillary incision 5-cm long at the 4^th^ intercostal space, entered the chest, and cardiopulmonary bypass was performed through the right femoral arteriovenous area.

**Fig. 1:**
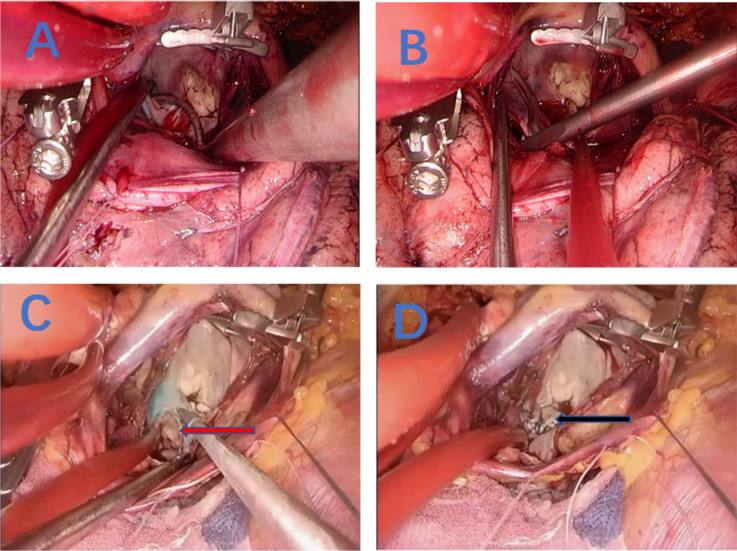
Left atrial ablation. (A) Ablation line from right inferior pulmonary vein to the left atrial appendage; (B) ablation line from right superior pulmonary vein to the left atrial appendage; (C) incision on the left pulmonary vein, marked by red arrow; (D) closing of the incision on the left pulmonary vein, marked by black arrow.

**Fig. 2:**
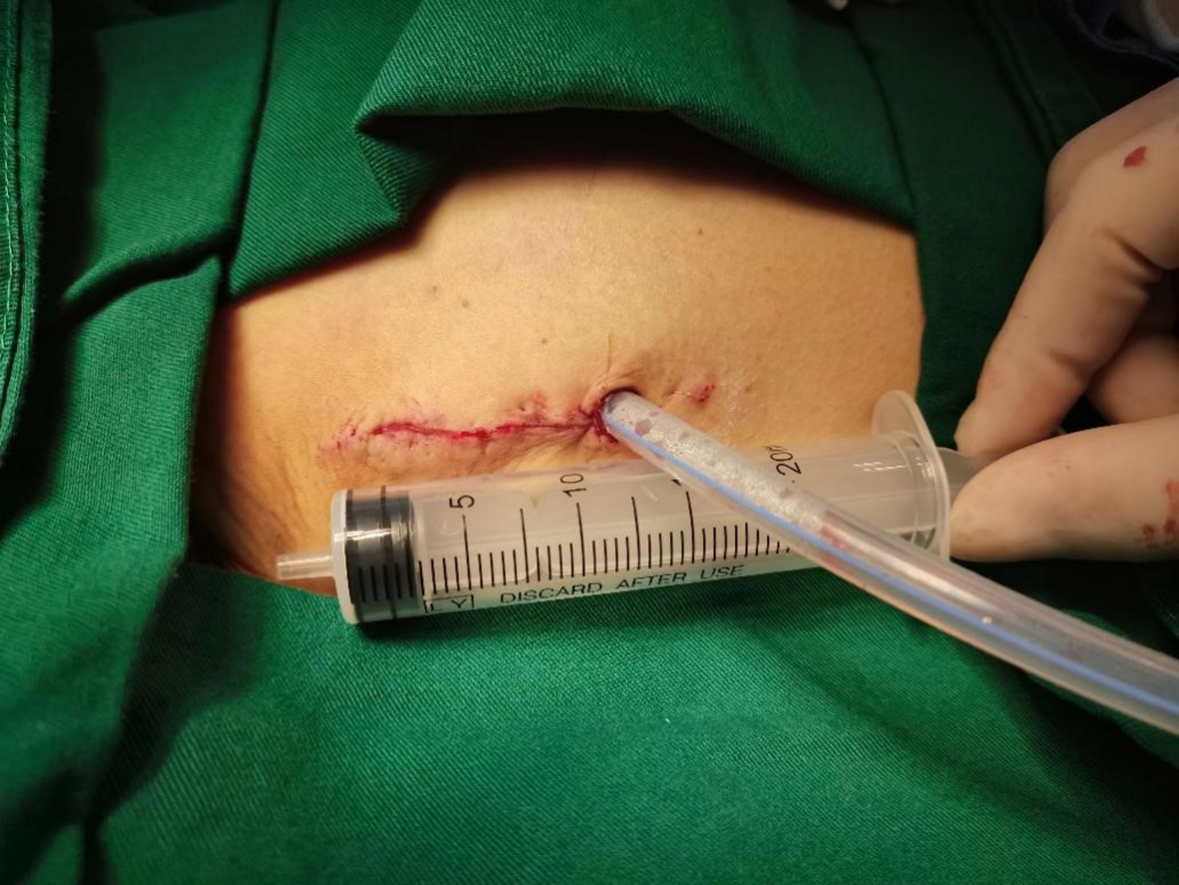
Surgical incision.

The aorta was clamped using a Glauber clamp (Cardio Vision® MIC Aortic Clamp, Cardio Medical GmbH, Germany), and the antegrade del Nido cardioplegia solution was administered. We started AF operation after the patient’s cardiac arrest, cut the left atrium along the atrial sulcus - the incision took a moderately longer time than in purely valve surgery -, and carried out circumferential right pulmonary vein ablation, right pulmonary vein to mitral valve circumferential ablation, left atrial inferior wall to left auricle ablation, left atrial roof to left atrial appendage ablation (Figure 1A), and left atrial appendage to left superior pulmonary vein ablation in a row; we cut the left atrial posterior wall between the left pulmonary vein and atrial appendage, with the incision connecting the left atrial superior and inferior walls and left atrial appendage ablation lines, to complete the circumferential left PVI - incision was 3-4 cm long (Figure 1C, red arrow) -, and closed the left pulmonary vein incision with 3-0 polypropylene sutures (Figure 1D) to build a complete isolation of the left atrial posterior wall (box lesion). We used a bipolar surgical ablation pen to perform complement ablation in the near mitral annulus, coronary sinus, and other places, and closed the left atrial appendage with interrupted suture to complete the left atrial lesion. We cut the right atrium from the right auricle to the inferior vena cava, and the incision stopped at the superior vena cava, inferior vena cava, and atrial sulcus; we performed a tricuspid annulus ablation, with a bipolar ablation pen at the tricuspid annulus and coronary venous sinus, to complete the right atrial lesion. The mitral valve procedure is the same with the previous procedure of our articles^[8]^. Subsequently, we closed the incisions in sequence (Figure 2).

### Ethical Approval

This study was approved by Zhejiang Provincial People’s Hospital Ethical Committee (QT2022408). All methods were performed in accordance with the relevant clinical guidelines and regulations.

## RESULTS

All patients finished the surgical operation smoothly, without perioperative death and complications such as secondary thoracotomy and vital organ insufficiency, and without permanent pacemaker implantation. Their median cardiopulmonary bypass time was 169 (interquartile range [IQR]: 109.75-202.75) minutes, aortic cross-clamping time was 106 (IQR: 77.75-125.50) minutes, ventilator assistance time was 6.5 (IQR: 0-10) hours, and eight of them had their endotracheal tubes removed immediately after surgery. Length of intensive care unit stay was 1.5 (IQR: 1-2) days, and 24-hour induced flow was 275.8±159.27 ml. Mitral valve replacements were performed in 12 cases, mechanical valve and biological valve in six cases each. Sixteen patients underwent mitral repair, nine patients underwent isolated mitral annuloplasty, and seven patients underwent artificial chordal replacement and mitral annuloplasty.

In terms of cardiac rhythm control, three patients were in a blanking period within three months after surgery, and according to the AF surgical treatment guidebook^[1,4]^, there was no need to recognize the ablation results. Two patients still had AF within three months after surgery, and one resumed sinus rhythm after electrical cardioversion therapy. The remaining 23 patients confirmed sinus rhythm by 24-hour Holter monitoring after stopping medication.

## DISCUSSION

AF is one of the common complications of mitral valve disease, which clearly increases the possibilities of postoperative embolism, long-term death, and cardiac insufficiency^[9,10]^. Maze procedure may substantially improve the patients’ long-term survival rate and life quality^[11-13]^. Currently, minimally invasive mitral valve surgery is substantially mature, which is also the most popular; for mitral valve disease plus AF patients, it is still difficult to treat mitral valve and AF simultaneously with this surgery, in particular to treat circumferential left pulmonary veins; currently, the procedure is mainly monopolar or freeze ablation, but nowadays there is no commercial freezing equipment in China, while the effect of monopolar ablation is not satisfactory^[1,6,14]^; therefore, for mitral valve disease plus AF patients, especially in current minimally invasive technique conditions, the importance of effective minimally invasive maze procedure is self-evident. Upon learning from the maze III surgical experience, we consider using cut-and-sew technique to replace bipolar PVI ablation that cannot be completed due to lack of equipment; our findings have preliminarily shown the efficacy of such a procedure, for example, only three patients were still in a blanking period, while 24 of the remaining 25 patients resumed sinus rhythm three months after surgery, and the recent surgical results were satisfactory. Although the cardiopulmonary bypass lasted for about three hours, there was no perioperative death, no major bleeding, or secondary surgery, and eight of the patients had their endotracheal tubes removed immediately after surgery, which preliminarily proved the safety of the surgery.

The completeness and transmurality of the ablation lines play an important role in the maintenance of sinus rhythm. Current views believe that in terms of transmurality, bipolar clamp ablation is better than endocardial monopolar ablation, and cut-and-sew technique is better than ablation technique. In particular, the quality of the left atrial box lesion has an important impact on the outcome. The creation of the left atrial box lesion using cut-and-sew technique may be non-inferior to Cox-maze to restore sinus rhythm^[15-19]^. In terms of surgical efficacy, both atria maze is better than left atrium maze procedure^[1,4]^; currently, most ablation lines in maze procedure can be completed with bipolar ablation equipment, but it’s still difficult to build the left pulmonary vein ablation line. In this surgery, the left atrium was cut beside the left pulmonary vein, for full electrical isolation, the incision should be intersected with the connecting lines of the left atrium box, or bipolar ablation was added to the incision and the connecting lines to ensure the integrity of the ablation line, being the major reason for the high sinus rate of these patients; the difficulty of this surgery was how to control bleeding effectively, because this zone would become invisible once the heart was filled again. Our method was to sew the incision with 3-0 polypropylene sutures from the inner side of the atrium, while solidifying the suspect bleeding points. None of the 28 patients in this group required secondary clamp due to bleeding; in case of bleeding that requires secondary clamp, the intermittent reinforcement suture in the left atrium will be an effective option.

Due to the tight schedule, only 28 operations in this procedure were done so far. Most subjects have good basic cardiac function, short AF history, and left atrium diameter < 6 cm, which may be one of the major reasons for the satisfactory efficacy of this group. Meanwhile, due to limited experience, we are not sure about the surgical taboos underlying this procedure, however, judging from our preliminary experience, this procedure is not recommended for patients aged > 70 years, body mass index ≥ 28 kg/m^2^, with severe pulmonary insufficiency and prior cardiac surgery, and requiring treatment of more than one heart valve at the same time.

### Limitations

Currently, the technology used in this study is limited to a specific population, and further expansion of indications will require accumulation of experience and validation of results. This study only provides short-term results, and longer follow-up is still needed to evaluate the long-term effects of AF ablation.

## CONCLUSION

The minimally invasive full maze procedure with cut-and-sew technique to build the left pulmonary vein ablation line is effective. In selected subjects, this procedure combined with the minimally invasive mitral valve procedure is safe, efficacious, minimally invasive, easy to accept, and allows a beautiful incision.
